# Supplementary material for: Culexarchaeia, a novel archaeal class of anaerobic generalists inhabiting geothermal environments
Source: ISME Commun. 2022 Sep 20;2:86. doi: 10.1038/s43705-022-00175-8 (PMC9723716; doi:10.1038/s43705-022-00175-8)
Supplement: Supplementary file 1 — Supplementary Information [file 43705_2022_175_MOESM1_ESM.pdf]

Supplementary Information

for

Culexarchaeia, a novel archaeal class of anaerobic generalists inhabiting geothermal environments

Anthony J. Kohtz, Zackary J. Jay, Mackenzie Lynes, Viola Krukenberg, Roland Hatzenpichler

Supplementary Files

File Name: Supplemental Files 1-8 (Excel file)

Supplementary File 1: Lists of single copy marker genes, genomes, and genome identifiers used in the phylogenomic analysis shown in Figure 1A and Supplementary Figure 1.

Supplementary File 2: Presence-absence patterns for individual genomes, with genes involved in central information-processing machinery.

Supplementary File 3: Full list of genes found in Culexarchaeia MAGs that were used to construct Figure 3.

Supplementary File 4: Full list of abbreviations used in Figure 3.

Supplementary File 5: Full list of IMG or NCBI accessions for the [NiFe] hydrogenase complexes depicted in Figure 4.

Supplementary File 6: Presence and absence of methanogenesis marker proteins in Culexarchaeia and Methanomethylicia (Verstraetearchaeota) MAGs.

Supplementary File 7: Metadata and geochemical data for YNP sites LCB-003 and LCB-024.

Supplementary File 8: Metadata for Culexarchaeia 16S rRNA genes in NCBI and IMG databases.

Supplementary Table 1 Temperature profiles recorded by Alvin's heat flow probe on December 24th, 2016, during dive 4872. This table illustrates the dynamics of the hydrothermal vent field in Guaymas Basin, which makes an exact determination of the temperature at time of sampling hard. To ensure stable placement of the temperature probe, the probe's disk had to be buried 2-3 cm in the sediment. The sediment sample from which the metagenome was retrieved was taken approximately 40-60 and 30-50 minutes after temperature profiles 1 and 2 were obtained, respectively. To not interfere with the integrity of the sample, the temperatures were taken 1-2 meters away from where the eventual sample was taken. The sample was collected from approximately half-way between where the two temperature profiles were taken. Geolocation of sample: 27° 00.684 N, 111° 24.266 W. Water depth: 2000 m. The temperatures most closely reflecting the *in situ* temperature range at the time of sampling are highlighted in bold (53.2 and 83.2 ºC).

	Heat-flow probe location 1		Heat-flow probe location 2		
	Time 16:33	**Time 16:36**	Time 16:44	Time 16:47	**Time 16:50**
Temp. 1 (7-8 cm)	76.4 °C	**83.2 °C**	38.6 °C	50.7 °C	**53.2 °C**
Temp. 2 (~17 cm)	98.3 °C	101.8 °C	58.4 °C	71.1 °C	73.3 °C
Temp. 3 (~27 cm)	104.2 °C	105.6 °C	74.9 °C	81.8 °C	82.8 °C
Temp. 4 (~37 cm)	107.4 °C	108.1 °C	88.2 °C	92.1 °C	93.1 °C
Temp. 5 (~47 cm)	107.2 °C	107.7 °C	98.1 °C	100.1 °C	101.3 °C

Supplementary Table 2 Proposed naming scheme for the Culexarchaeia MAGs used in this study. Family, genus, and species designations were decided by considering our phylogenomic tree (Figure 1A), pairwise 16S rRNA gene nucleotide identities, and pairwise MAG AAI values. *, indicates MAG ID used to designate type species.

<table>
<tr><th>Phylum</th><th>Class</th><th>Order</th><th>Family</th><th>Genus</th><th>Species</th><th>MAG ID</th></tr>
<tr><td rowspan="10">Culexarchaeota (NCBI) / Thermoproteota (GTDB)</td><td rowspan="10">Culexarchaeia</td><td rowspan="10">Culexarchaeles</td><td rowspan="6">Culexarchaeaceae</td><td rowspan="6">Culexarchaeum</td><td rowspan="4">yellowstonense</td><td>YNP-LCB-24-027 *</td></tr>
<tr><td>YNP-LCB-3-016</td></tr>
<tr><td>YNP-WB-040</td></tr>
<tr><td>YNP-WB-062</td></tr>
<tr><td>jinzeense</td><td>JZ-Bin-30 *</td></tr>
<tr><td>nevadense</td><td>GBS-70-058 *</td></tr>
<tr><td rowspan="4">Culexmicrobiaceae</td><td rowspan="4">Culexmicrobium</td><td>cathedralense</td><td>GB-1867-005 *</td></tr>
<tr><td>profundum</td><td>GB-1867-035 *</td></tr>
<tr><td rowspan="2">thermophilum</td><td>GB-1845-036 *</td></tr>
<tr><td>GB-1867-097</td></tr>
</table>

Supplementary figures

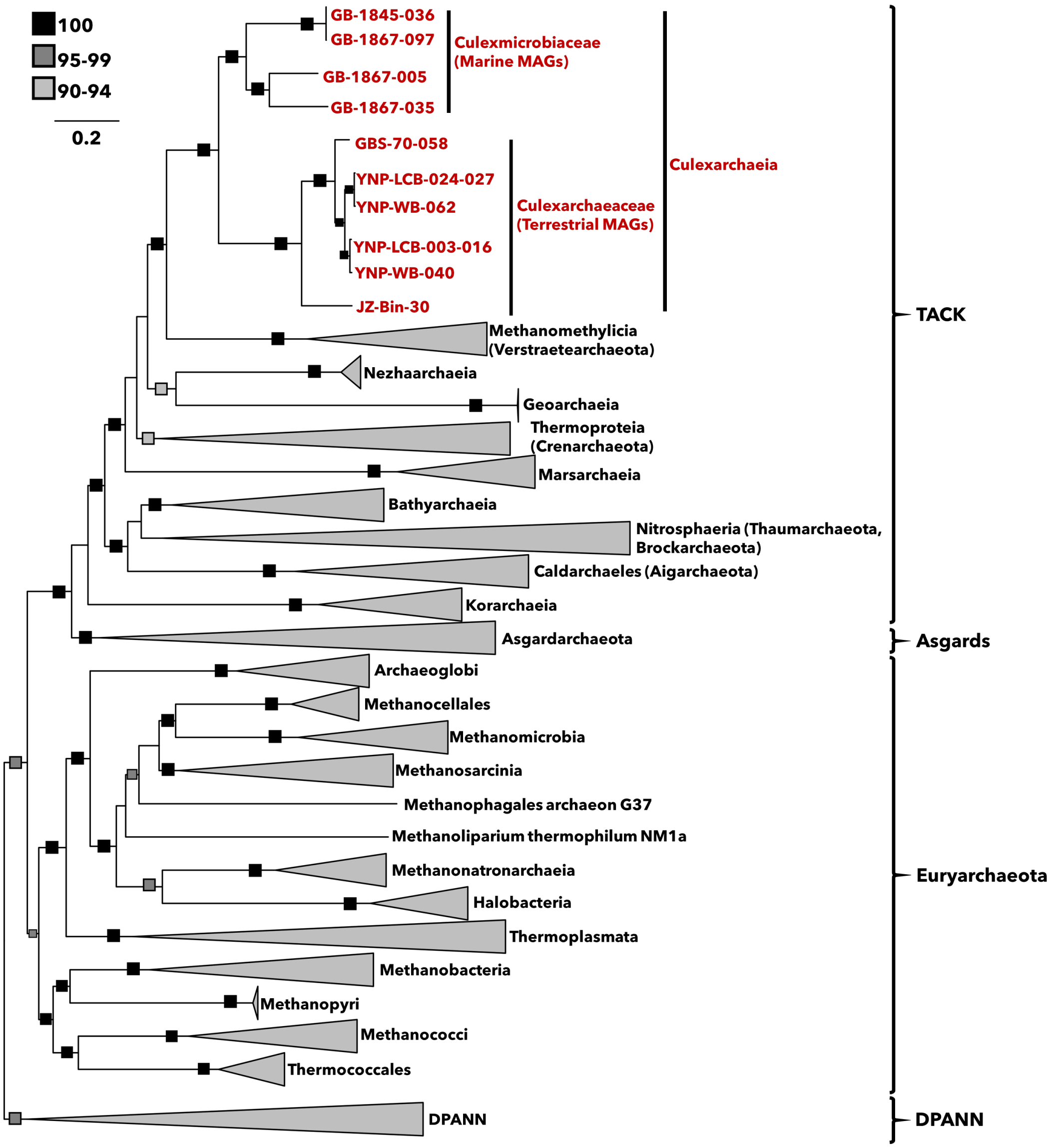

Supplementary Figure 1 Phylogenomic tree of Culexarchaeia MAGs and reference archaeal genomes. Maximum-likelihood tree, inferred with IQtree and the best-fit LG+C60+F+G model, using a concatenated set of 46 conserved ribosomal proteins (Supplementary File 1). Ultrafast bootstrap support values of 100, 95-99, and 90-94 are indicated with black, dark gray, and light gray squares, respectively.

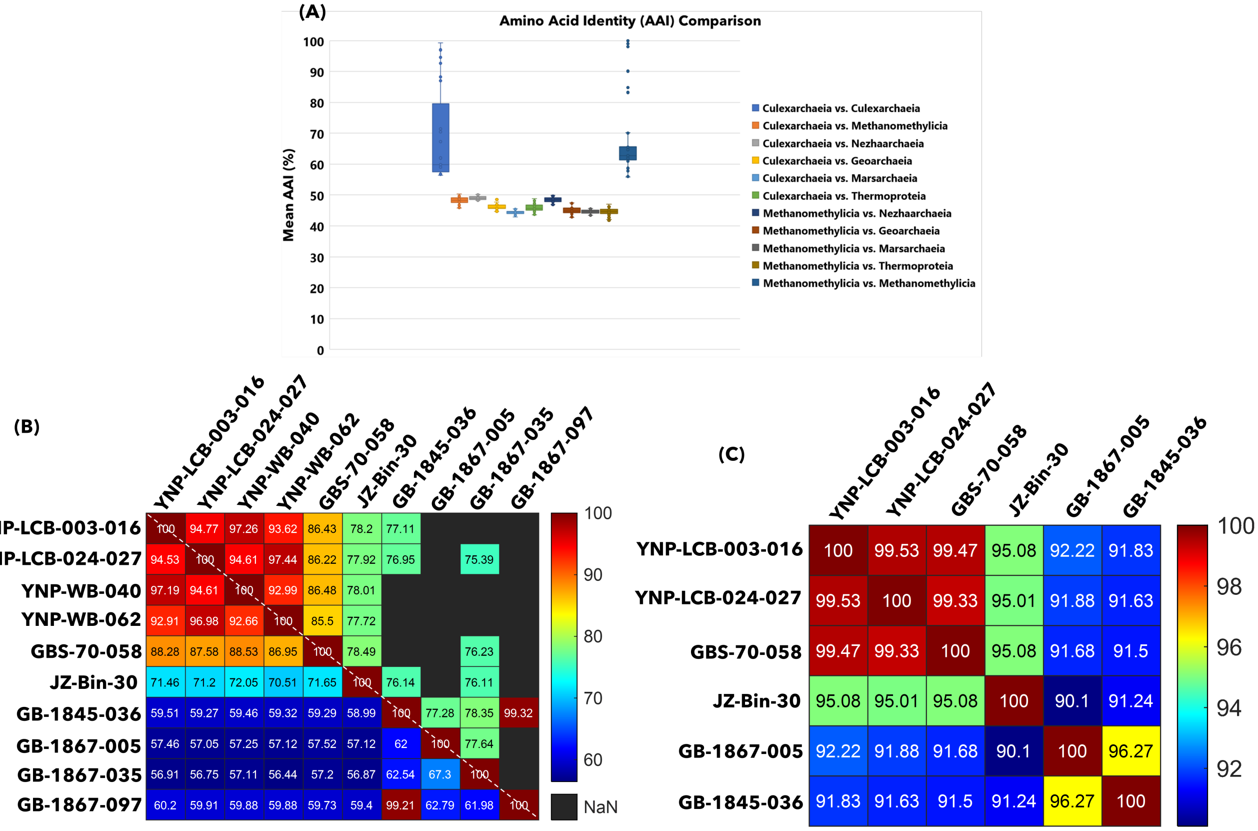

Supplementary Figure 2 Amino acid identities (AAI) of Culexarchaeia and major TACK lineages. Intra-clade AAI, ANI, and 16S rRNA gene percent identity of Culexarchaeia. (A) Analysis shows that the average AAI between TACK classes is below 50%, consistent with the designation of Culexarchaeia as a class level lineage within the TACK superphylum. Thick bar, median; first and third quartile, upper and lower bounds of the box, respectively. Upper and lower whiskers extend to the highest and lowest values within 1.5x of the interquartile range, respectively. **(B)** Intra-clade AAI and ANI of Culexarchaeia. Values above and below the dashed white line represent ANI and AAI values, respectively. AAI values were calculated with compareM and ANI values were calculated with fastANI. **(C)** Pairwise % identity of Culexarchaeia 16S rRNA gene sequences calculated by BLASTn.

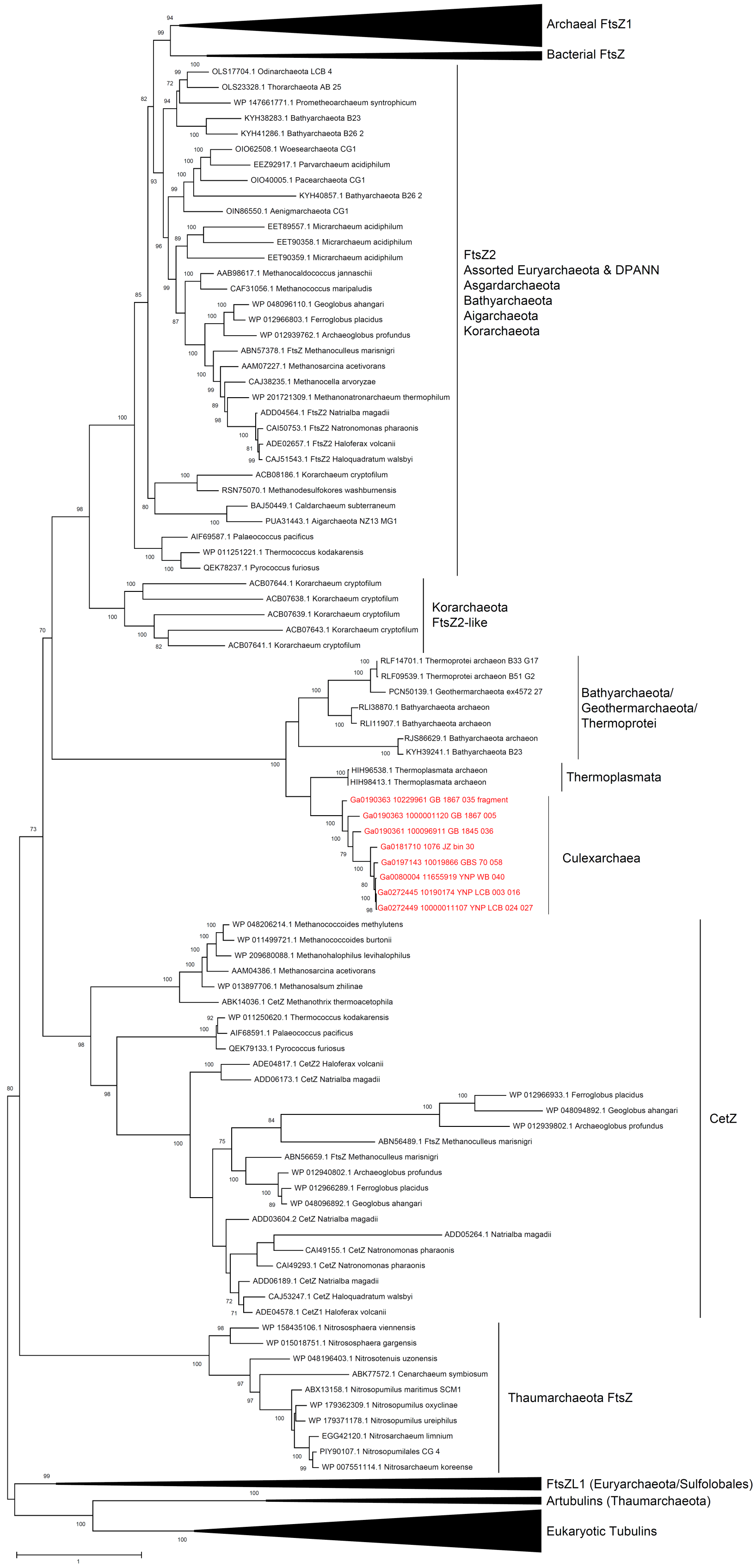

Supplementary Figure 3 Maximum likelihood phylogeny of tubulin superfamily proteins. Amino acid sequences were aligned using Mafft-LINSi and trimmed with a 70% gap threshold using trimal. Tree was constructed with IQtree2 and the best fit model LG+R5. Ultrafast bootstrap support values above 70 are indicated at each node, and values below 70 are not displayed. Culexarchaeia FtsZ-like sequences are highlighted in red.

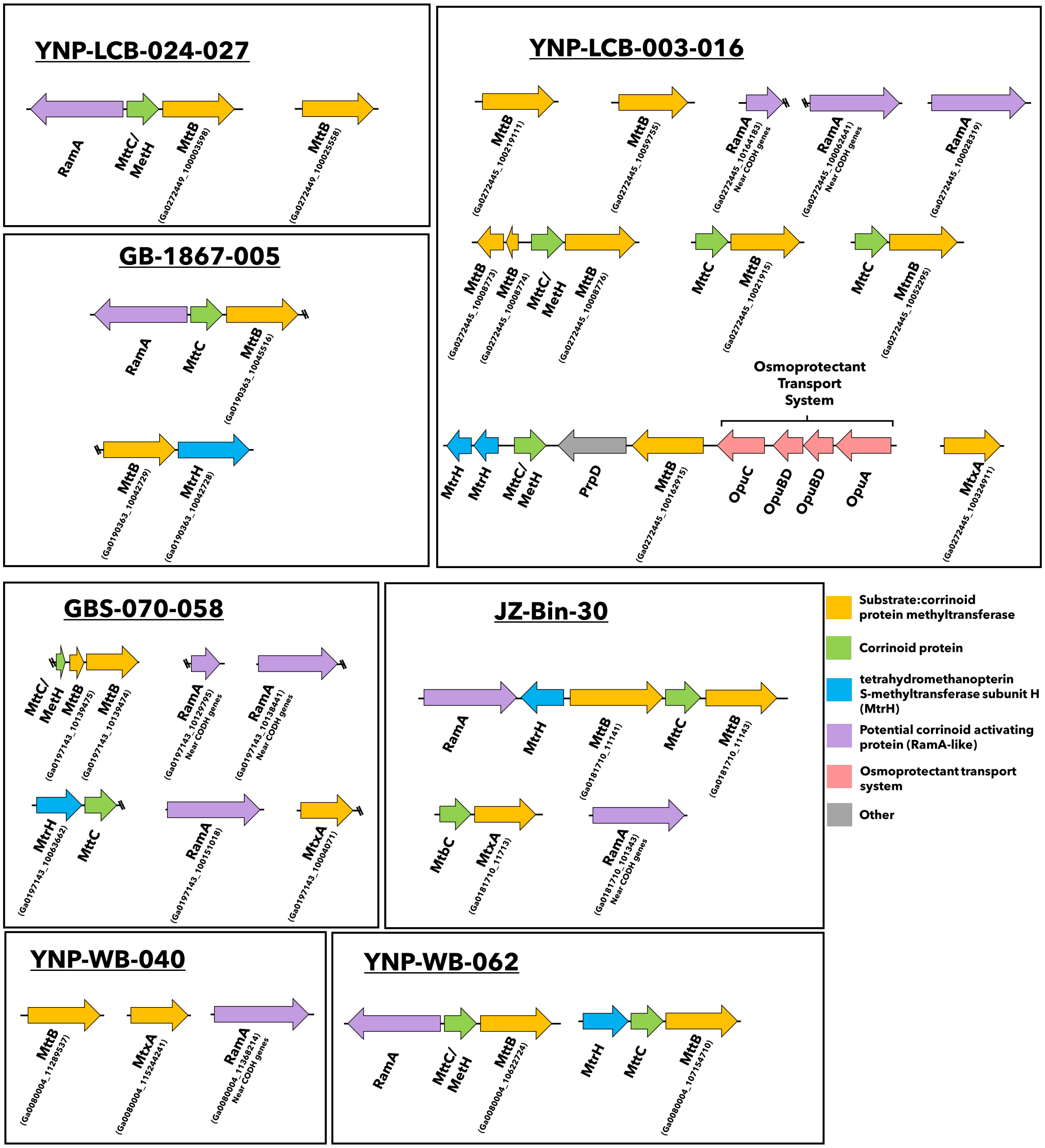

Supplementary Figure 4 Organization of Culexarchaeia methyltransferase genes. The color of the arrows corresponds to the predicted gene function listed. Some proteins in the family COG3984 (RamA-like) were found located next to carbon monoxide dehydrogenase (CODH) genes, suggesting they may play a role in the function of this complex instead. MAGs not listed did not have any methyltransferase genes encoded. Double slashes indicate the gene is truncated at the end of a scaffold. Each cluster of arrows depicted represents a separate scaffold. Locus tag(s) for genes on IMG are indicated in parentheses for each scaffold.

Supplementary Methods

Cell extractions and catalyzed reporter deposition fluorescence *in situ* hybridization (CARD-FISH)

Sediment samples from YNP sites LCB-003 and LCB-024 were collected in July 2019, fixed with paraformaldehyde at a final concentration of 2% for 8 h at 4°C, washed twice with 1x phosphate buffered saline (PBS) and stored in 1x PBS at 4 °C until cells were extracted. Extractions were performed by diluting aliquots of the sediment slurries 1:1 in PBS, adding methanol to a final concentration of 10%, Tween-20 to a final concentration of 0.1%, and vortexing the solution horizontally (Vortex-Genie2, Scientific Industries, Inc., Bohemia, NY) for five minutes to detach cells from particles. After vortexing, an equal volume of 80% (w/v) Nycodenz was layered under the sample and centrifuged at 16,000 g for 30 min at 4°C. Following centrifugation, the supernatant and interphase layers were transferred to a new tube, diluted 1:1 in PBS, and centrifuged at 16,000 g for 5 min to pellet the cells. The supernatant was removed, and the cells were resuspended in 1x PBS and stored at 4°C. Aliquots of the cell suspension were immobilized onto glass slides and CARD-FISH was performed as described in (1) with the following modifications. Cell permeabilization was attempted with either proteinase K (15 µg/mL) for 10 minutes or with 0.1 M HCl for 1 minute at room temperature. Endogenous peroxidases were inactivated by incubating in 0.01 M HCl for 15 minutes. A Culexarchaeaceae-specific probe, Culex824 (5'-TCCACCTAACACCTAGCC-3'), targeted at the 16S rRNA of Culexarchaeaceae found in terrestrial hot springs, was designed using the probe design tool within the Arb software and the Silva 132 reference database (2, 3). Hybridization of probe Culex824 was attempted at formamide concentrations 0, 10, 20, and 35 %. Positive and negative control hybridization reactions were performed with the general archaeal probe Arc915 (5'-GTGCTCCCCCGCCAATTCCT-3') and NON338 (5'-ACTCCTACGGGAGGCAGC-3'), respectively (4). All probes were labeled with horseradish peroxidase (HRP) and were purchased from Biomers (Ulm, Germany). Tyramide signal amplification was performed with Alexa Fluor 594 (Thermo Fisher). Samples were stained with DAPI, embedded in citifluor, and visualized under an epifluorescence microscope (Leica DM4B).

Supplementary results and discussion

Reclassification of JZ-bin-30

Based on our analyses, we propose to reclassify bin JZ-bin-30, originally given the provisional taxonomic assignment *Candidatus* Methanomedium jinzeense (5), as *Candidatus* Culexarchaeum jinzeense sp. nov. (etymology below). We base this re-assignment on the observation in 16S rRNA gene and phylogenomic analyses that JZ-bin-30 does not fall within the candidate class Methanomethylicia, but rather within the new candidate class Culexarchaeia. Additionally, in contrast to what the name Methanomedium implies,

all currently available Culexarchaeia MAGs, including JZ-bin-30, lack the key enzyme for methanogenesis (methyl-coenzyme M reductase; Mcr) and lack methanogenesis marker genes that have been identified in both *bona fide* Euryarchaeotal methanogens and newly proposed MCR-encoding archaea outside the Euryarchaeota (6).

References

1. Hatzenpichler R, Lebedeva EV, Spieck E, Stoecker K, Richter A, Daims H, Wagner M. 2008. A moderately thermophilic ammonia-oxidizing crenarchaeote from a hot spring. Proceedings of the National Academy of Sciences 105:2134-2139.
2. Quast C, Pruesse E, Yilmaz P, Gerken J, Schweer T, Yarza P, Peplies J, Glöckner FO. 2012. The SILVA ribosomal RNA gene database project: improved data processing and web-based tools. Nucleic acids research 41:D590-D596.
3. Ludwig W, Strunk O, Westram R, Richter L, Meier H, Yadhukumar, Buchner A, Lai T, Steppi S, Jobb G. 2004. ARB: a software environment for sequence data. Nucleic acids research 32:1363-1371.
4. Ravenschlag K, Sahm K, Amann R. 2001. Quantitative molecular analysis of the microbial community in marine Arctic sediments (Svalbard). Applied and Environmental Microbiology 67:387-395.
5. Berghuis BA, Yu FB, Schulz F, Blainey PC, Woyke T, Quake SR. 2019. Hydrogenotrophic methanogenesis in archaeal phylum Verstraetearchaeota reveals the shared ancestry of all methanogens. Proceedings of the National Academy of Sciences 116:5037-5044.
6. Borrel G, Adam PS, McKay LJ, Chen L-X, Sierra-García IN, Sieber CM, Letourneur Q, Ghozlane A, Andersen GL, Li W-J. 2019. Wide diversity of methane and short-chain alkane metabolisms in uncultured archaea. Nature microbiology 4:603-613.
